# Advancing Diabetic Care Through Non-Invasive Glucose Monitoring Using Optical Sensors and IoT Technologies

**DOI:** 10.12688/f1000research.173871.2

**Published:** 2026-05-05

**Authors:** Hanaa S. Basheer, Anes A. Al-Sharqi

**Affiliations:** 1 Photonics unit, University of Baghdad, Institute of Laser for Postgraduate Studies, Baghdad, Iraq

**Keywords:** CGM, diabetic, IoT, non-invasive BGM technologies, Markov chain

## Abstract

Diabetic Patients must monitor their Blood Glucose (BG) continually to control their glycemia at least twice a day using a finger prick. Patients visit a laboratory every three months for glycated hemoglobin (HbA1c). According to the World Health Organization (WHO), the constitution of Iraqi diabetes patients is 13.9%. An Internet of Things (IoT) based framework for non-invasive BG monitoring is recently developed. We aim to enhance the Continuous Glucose Monitoring (CGM).

Starting with a review of IoT technologies and some of CGM commercial devices recommended by ISO 15197 standard. A questionnaire sheet is distributed to determine how much patients in Iraq knew about their health situation and whether they are interested in using new IoT technologies. Women patients are volunteered to check their BG using both finger prick (FP) and a CGM device for comparing. The suggested method is to connect CGM to a smart device to show alarm messages when needed.

The results show how important to introduce patients about new technologies. A CGM and FP results are checked for similarity using statistical package. The findings demonstrated that, significantly at P < 0.05, there were no differences between both methods based on the standard Ambulatory Glucose Profile (AGP) report.

This study shows how Iraqi’s patients feel when using a CGM and new IoT technologies. CGM output is accurate but appeared every 15 min which may be uncomfortable. A method is suggested to transfer CGM output to a smart device to be controlled by algorithms, where an alarm message is showed every 8 h when BG is normal or a colored alarm will appear. GMI% will be calculated every two weeks using data stored in the cloud to estimate HbA1c level depending on Markov chain. This figures how BG changes in a shorter timeframe, helping in fine-tune diabetes management plans.

## I. Introduction

Lifestyle medicine is the application of evidence-based lifestyle approaches to prevent and treat continuing diseases, such as diabetes. Lifestyle Medicine leads patients to live healthier by following the six advice which are: “eating a predominantly whole food, a plant-based diet, Regular physical activity, Adequate sleep, Stress management, avoidance of risky substance use, and positive social connections”. ^1^ According to the WHO a proportion of Iraqi diabetes patients is 13.9%.

The latest technologies make a huge change in monitoring a patient’s situation with less pain, less time and less cost. In the 1970s, systems were developed to provide accurate results of the glucose level based on blood samples. This technology, called the Self-Monitoring of Blood Glucose (SMBG), allows patients with diabetes to monitor his/her glucose levels daily. Although SMBGs systems have highly predicted results, they are still painful and uncomfortable to use because they are based on fingerpicks for each test. In the 1980s, companies began to develop new devices based on CGM using non-blood tests. ^2^ In the 1990s, IoT healthcare was launched, and it has the ability to connect different devices that provide services through the Internet in real time. These IoT devices can be household objects or appliances, and are all called “things.” Things can collect and analyze data according to the required services. People with diabetes can live vital lives if they regularly monitor their glucose levels and keep them in range. Diabetes management strategies include education, physical activity, nutrition, medication, and lifestyle management. ^3^ The development of a self-aware patient agent (SPA) within a mobile agent environment was suggested in. ^4^

An important work was presented in 2019 showing how an auto check of glucose level for a patient can be performed using a biological sensor that can be sent through the internet to the doctor. The proposal was implemented and instrumented as an IoT system, in which all the measurements were carried out in vivo to show good benefits for patients since they follow up their conditions with less cost and painless. Its limitation is the use of bulky instruments that cannot be used during daily activities. ^5^ HbA1C is to measures the average amount of sugar in the blood over the past few months; for diabetic patients, the goal is to reach 7%, so this does not mean that their condition went away, but it means that their blood sugar is well managed. ^6^ One of the limitations of self-glucose testing is the absence of stability in (HbA1C), which is a very important parameter because it affects the nutrition plan, physical activity, and/or medication treatment. With all existing BG test tools, patients should be aware of medications that can interfere with glucometer accuracy. In 2021, a study of the correlation between vitamin D, fasting glucose, and A1c showed that the results were not significant. ^7– 9^ Another study showed a significant association between BG level control and (age, education level, monthly income). ^10^ Technologies with education, follow-up, and care can improve the lives and health of people with diabetes. ^11^ Many techniques have been introduced for individual use, such as carbon nanotubes and plasmonics, but these are still ideas despite progress on the theoretical side. ^12^ For example, in 2022, a method based on a plasmonic nanostructure glucose sensor and gold–silver core–shell nanoparticles as the sensing platform were developed. The oxidative etching of the silver shell and the concentration of hydrogen peroxide and glucose can be determined via spectral changes, that’s why this approach can be used for diabetes diagnosis and health monitoring. ^13^ Another example was presented, which used a Multilayer Neural Network trained on data from a dataset of Iraqi diabetes patients obtained from the Specialized Center for Endocrine Glands and Diabetes Diseases. ^14^

A work that invented a high-Q microwave sensor device for non-invasive real time measurement of glucose was published in 2021. This device is to detect low and high glucose levels by injecting liquid through a fluidic channel over the microwave sensor which equipped with an active feedback loop that can be used to compensate for the media serum loss and accurately. The authors claim that their non-invasive frequency shift-based system is the first to detect the glucose levels, which correspond to human physiological glucose levels, using active components but without mentioning the final design for the device to be used by human. ^15^ In 2023 a microwave planar sensing platform is proposed. It has a monitoring system composed of a machine learning algorithm coupled with a flexible microwave sensor. The fingertip is scanned on the planar sensor, which enables deeper interrogation of the skin layers through the wave propagation. The microwave sensor size is very small to fully covered with a single index finger and suitable for human body loading conditions. A sensor is attached to four volunteers which asked to stay indoors with limited movement of their hands where data is recorded every 5 s for eight days, 3 h a day. An AccuChek glucometer is used also to measure the GL every 10 min to check the proposed sensor performance. The results confirmed a high correlation between both sensors. Moreover, the authors predicted a neural network to enhanced the sensor performance by taking the real time changes in the environments in to consideration. ^16^

The percentage of Iraqi diabetes patients is 13.9% and may increase by 54% by 2030; therefore, there is a need to educate patients about the importance of using IoT devices become a must by using efficient experiments, which is one of the goals of this work. There are two types of diabetes technologies, CGM and BGM. General principles for CGM devices must be considered for patients on oral medicine before use, which are ^7^:

• Selecting a device should be based on a person’s specific needs and skill level.
• Ensure that the person receives education and training about the device before using.
• People using CGM should continue with a third party such as a diabetes center for regular follow-ups.
  Nowadays, many techniques are developed to be simple, less painful, and non-invasive techniques for measuring blood glucose levels. Non-invasive techniques can be divided into glucose oxidase, optical spectroscopy, and dielectric spectroscopy. ^17^
  The aim of this research is to use IoT technologies to enhance CGM devices by (1) reducing alarm beats to the minimum or buzz, when necessary, (2) calculating A1C every two weeks that reflects the overall glucose control, and helps monitor treatment effectiveness, and (3) estimating A1C for the next 14 days to show how a patient knows how his lifestyle affects his BG.

## II. Background

Optical approaches are important for BG because they offer non-invasive, painless, and continuous monitoring alternatives to traditional finger-prick methods. In this section, a brief review of optical approaches is presented. Moreover, a review of the latest CGM commercial devices recommended by the ISO 15197 standard provides quality guidelines.

### A. Optical approaches for Non-invasive glucose level monitoring

**Figure 1.**
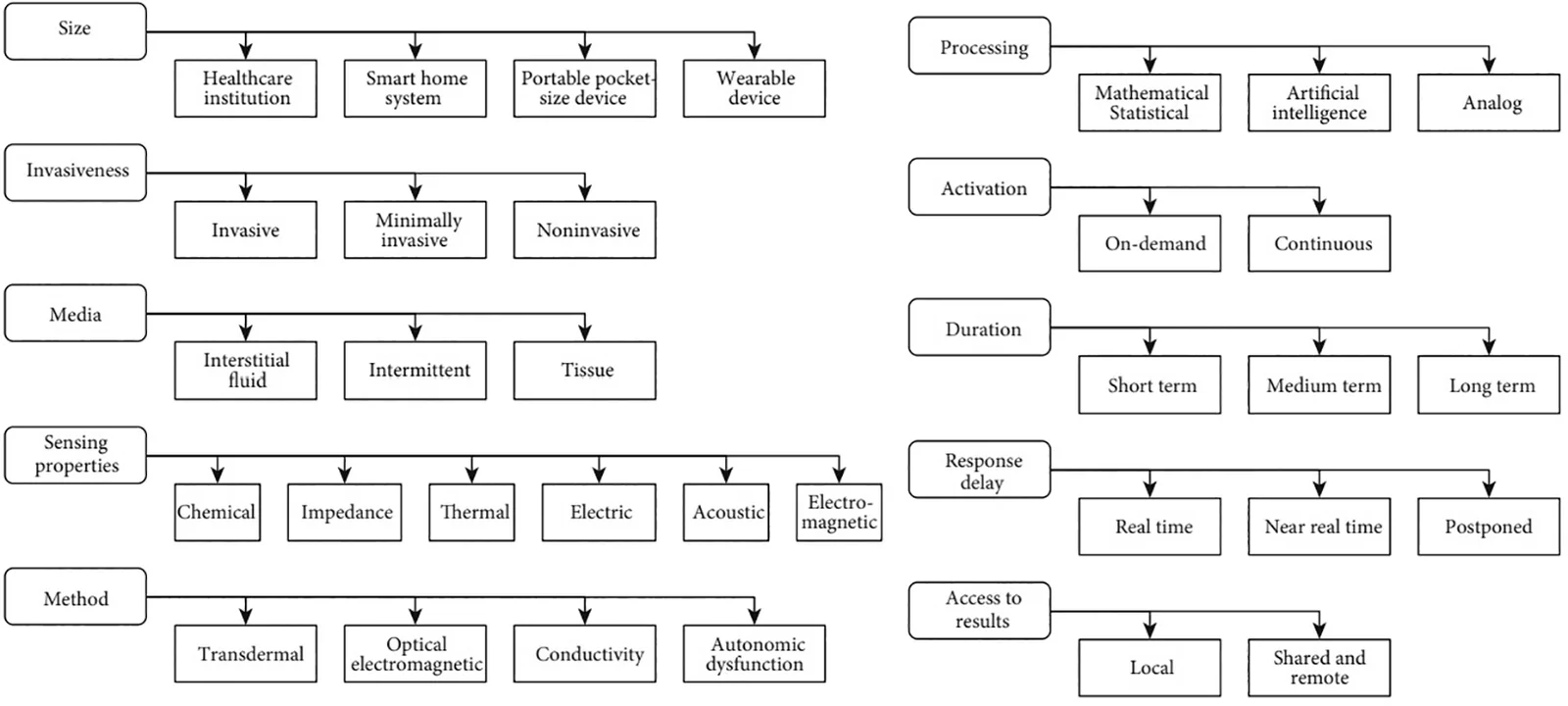
Classification of glucose measurements according to different criteria. ^19^

The standard SMBG test has a limitation because the blood sample gives only a fragment of the real glucose level discontinuously, meaning that SMBG cannot indicate the ongoing glucose level changes during the day, even if this test is performed frequently. Changing symptomatic hypoglycemia or hyperglycemia for a patient could be uncomfortable and make it difficult to make a correct healthcare decision. For this reason, a recent study ensured that many glucometers for self-monitoring do not meet the level of accuracy; therefore, focusing on using Continuous Glucose Monitoring (CGM) is highly considered to help a patient decide on the medicine doses. CGM provides glucose concentration measurements based on a skin sensor that measures glucose concentration in the interstitial fluid and transmits the sensor values (usually at 5–15 min intervals) to a dedicated receiver, which can be seen in real time. ^18^ A classification for glucose level monitoring is presented in reference ^19^ based on different criteria, as shown in Figure 1. Non-invasive glucose level monitoring methods are based on measuring glucose concentration based on its chemical, thermal, electrical, or optical sensing properties.

In 2021, a review of non-Invasive sensing systems for detecting glucose levels revealed a classification for the existing methods according to the sensor types. The classes include non-invasive glucose detection through the patient’s eye, finger, wrist, forearm, and abdomen. ^20^ Light is focused on biological tissues and is affected by reflection, scattering, and transmission, according to the structure and chemical components of the tissue sample. This is the concept behind optical-based non-invasive glucose measurement methods. These properties are differentiated according to the analysis of the device used; thus, the interpretation of the glucose levels is based on the received spectrum. Many optical non-invasive methods have been analyzed to successfully test glucose levels and are classified in references. ^19, 21^ Another classification based on optical approaches divides the classes into electromechanical, electrochemical, and electromagnetic (EM) methods. This study discusses the characteristics required for efficient sensors to predict a correct decision from the collected data. The authors clarified the optical approaches based on many features. ^22^

In 2021, a design was presented to test BG using optical non-invasive sensors based on finger tissue, including an infrared light-emitting diode (LED) with a wavelength of 950 nm, an ultrasound sender with 2 MHz, a sensitive IR detector, and two “Arduino” microcontrollers. The implementation of the system methodology depends on spreading the near-infrared light through the finger to obtain the glucose level. The authors did not provide the patient with the facility for auto use, as did other designs. ^23^ Near-infrared NIR with LED is one of the technologies used with the associated sensor circuit to monitor the BG concentration. The experimental results of this approach and a mathematical model showed that the higher the glucose concentration, the lower the sensor output voltage. As the authors explained, this approach can be beneficial for individual patients by reducing the need for finger pricking and the pain it causes; however, further studies are needed to evaluate the accuracy and reliability of the designed system under different conditions. ^24^ In 2023, a study considering IoT for non-invasive BG was published. The work idea is to build a system consisting of LED and NIR sensors to produce signals that are disseminated through the fingertip. A phototransistor is positioned next to the LED to detect any reflected signals and be stored in a “Thing-speak.” Mathematically analyzing the reflected signal intensity with an algorithm installed in the “Arduino” shows the relationship between glucose concentration and voltage. The experimental results of this study showed that the accuracy ranged from 1.13 to 16.41%. Another study showed that LED light and biosensors provide good results for measuring individual glucose levels. More research is required to develop non-invasive BGL systems. There is still a need to improve the hardware of the devices because most IR technologies are software. Developing a design with low power and cost that is processed in real time would lead to a non-invasive system with high performance. ^21^ A method of Fast IR spectroscopic glucose concentration measurement using a mid-infrared wavelength-swept pulsed quantum cascade laser (QCL) is adopted to measure the concentration of blood glucose using attenuated total reflection. The high power of the QCL with the proposed algorithm enables the BG measurements. The proposed method allows detection of the skin and enables the measurement of the tear film on the eyeball in the tangential direction. This has potential applications in the diagnosis and management of diabetes and minimizes the measurement time to 20 ms. ^25^

In Conclusion, different CGM methods are depending on the sensor types that were used. The concept behind optical-based non-invasive glucose measurement methods is the light focused on biological tissues, where its reflection, scattering, and transmission are the parameters for BG measurement. Optical approaches use either spreading near-infrared light, LED and NIR sensors, LED land biosensors, or fast IR spectroscopic and mid-infrared wavelength. 

### B. Commercial devices for non-invasive glucose level monitoring

“The standard ISO 15197 provides the quality guidelines, requirements, and specifications that glucose measuring devices should comply with to guarantee their suitability for human use”. The ISO guidelines evaluate each device and show whether it is suitable for commercialization. ^12^ Many commercial devices in the market are approved by the ISO standard, and almost all of them use spectroscopic techniques, especially NIR. A review of some of these devices, especially those using IoT technologies.

**Figure 2.**
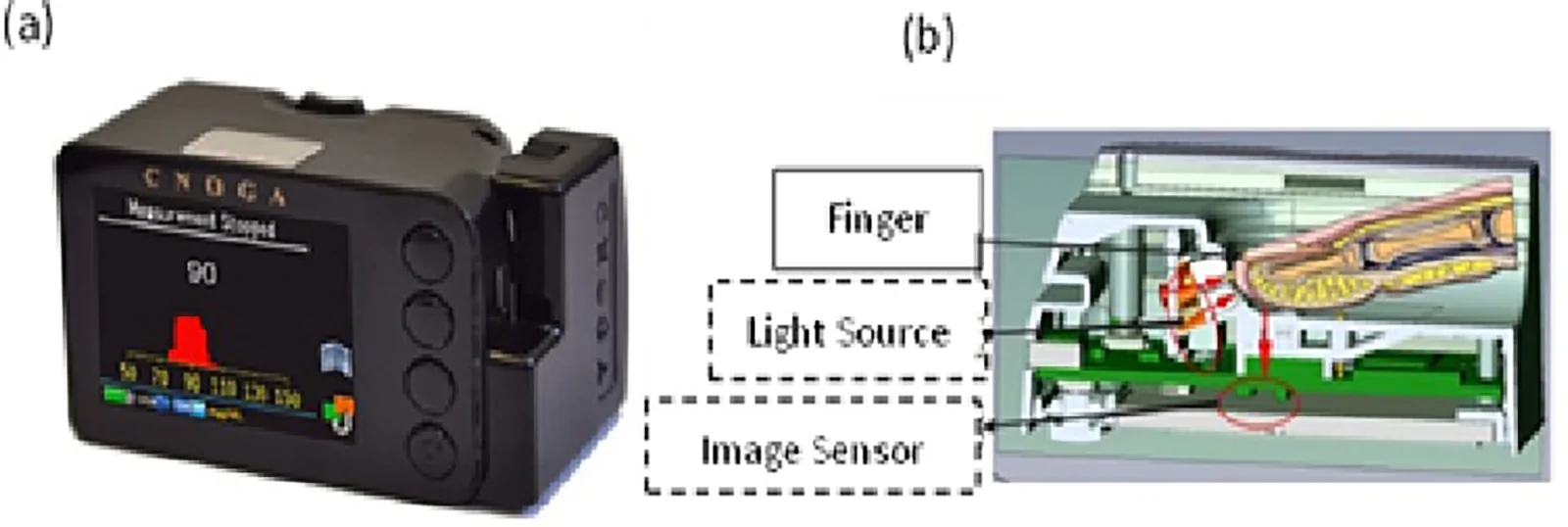
(a) The CoG device, (b) A cross sectional view of the CoG. ^26^

**Figure 3.**
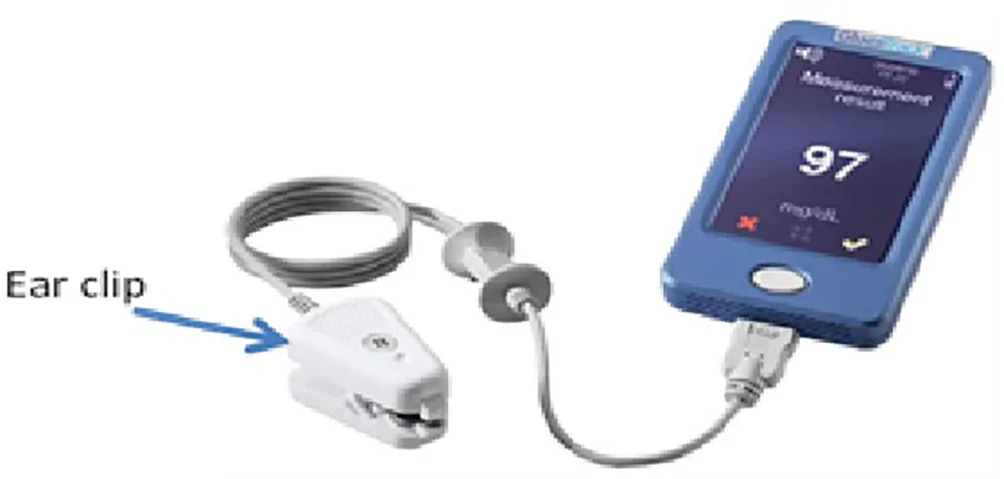
Invasive blood glucose meter GlucoTrack. ^29^

**Figure 4.**
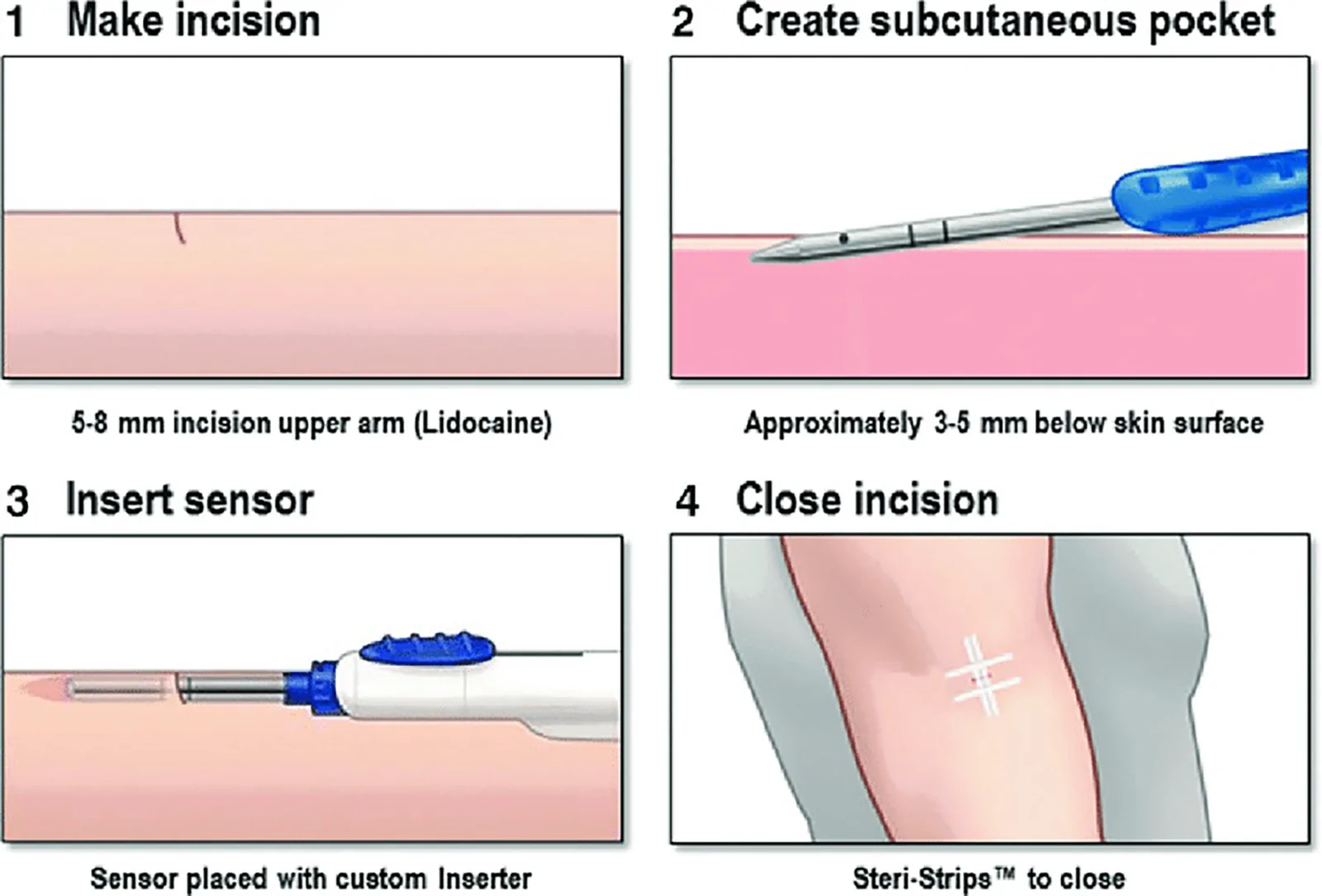
Eversion sensor insertion procedure. ^28^

**Figure 5.**
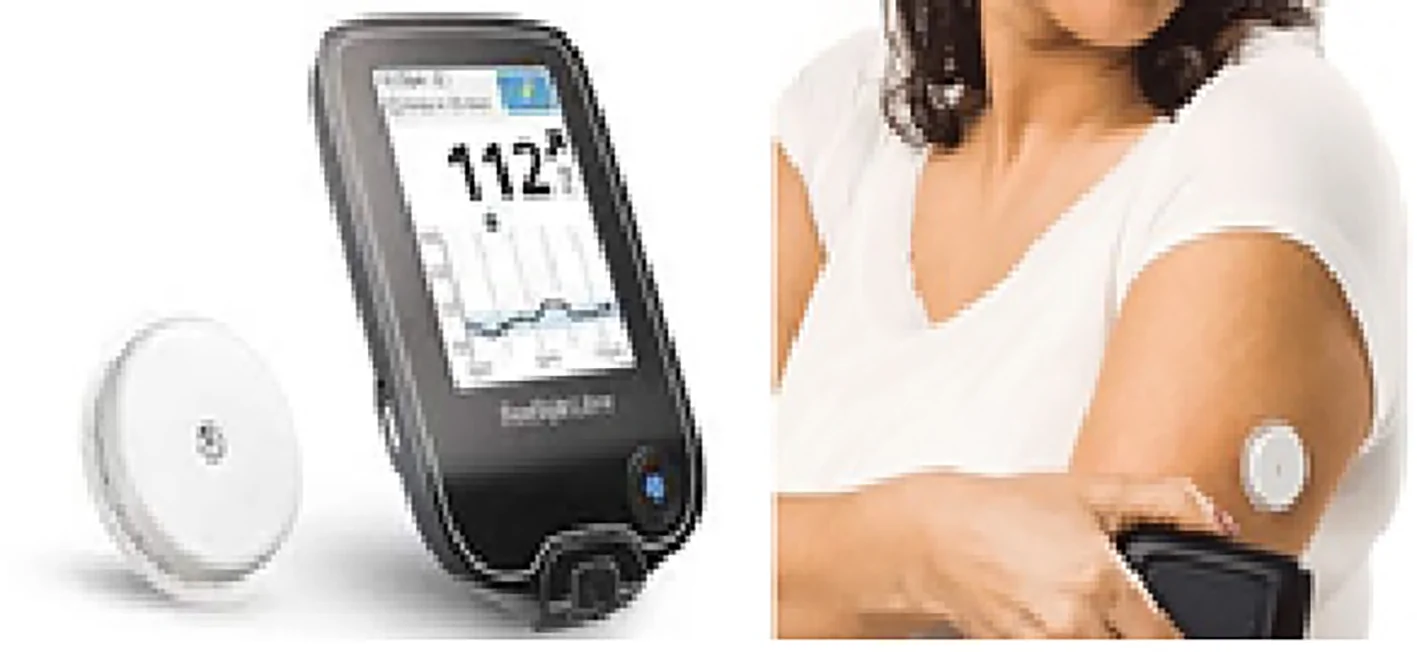
Abbott lifestyle, and attached skin sensor. ^32^

- Non-invasive blood glucose meter (Combo Glucometer) CoG: This device is for invasive and non-invasive glucose tests, it is small, lightweight, and easy to hold. The device technology consists of a color image sensor, four LEDs (IR spectrum from 625 to 940 nm), and a digital signal processor (DSP). The control part consisted of four touch buttons, a display, an audio speaker, and a microcontroller. This device includes process management, storage, and power management. The way of using can be shown in Figure 2, the traverse light is diffused over the image sensor range, and therefore all three colors (red, green, and blue) will sense the traverse light, each one with a different sensing value and will be stored in the memory buffer. Computation was then performed using the algorithm in the DSP. ^26^
- GlucoTrack (Integrity Applications): The device shown in Figure 3 automatically uses personal ear clip (PEC) sensors to measure the three parameters of the earlobe tissue: ultrasonic, electromagnetic, and thermal. These parameters were found to be due to glucose-related shifts in ion concentration, density, compressibility, and hydration of both cellular and extracellular compartments of the tissue. This device is used in patients with prediabetes and type 2 diabetes mellitus. A clinical trial conducted on 17 subjects showed that 98.0% of the readings were acceptable. ^27^
- Eversense (Senseonics): This device is non-invasive and uses fluorescent light. However, the fact that the device needs to be under the skin and its lifetime is limited to 180 days means that additional research is required to prolong the life of the sensor. ^12^ Figure 4 illustrates how the sensor was inserted under the skin. ^28^
- Abbott Freestyle Library (CGM): This device has a handheld reader and a disposable sensor that is attached to the arm. The patient scanned the attached sensor with a meter for glucose reading, as shown in Figure 5. The meter has built-in BG and ketone meters suitable for FreeStyle Optium blood glucose and blood ketone test strips. ^30^ A comparison by Galindo, R.J. and his team were made between FreeStyle CGM and a bedside point-of-care capillary glucose testing (POC). The results showed leaning for lower glucose concentrations in the CGM results. However, its clinical accuracy was acceptable. CGM detected more nocturnal and prolonged hypoglycemic episodes compared with limited daily testing with POC, indicating that this approach has failed to detect these episodes efficiently. Conclusion This study showed a good correlation between CGM taken by an Abbott lifestyle sensor and laboratory device for testing glucose values. ^31^ This device can also be connected to the person’s smart mobile using Bluetooth to give the patient a regular alarm about his glucose level, which automatically collects sensor glucose that provides a good indication of BG values every 15 min, but can only store 8 h of data.

IoT is considered a promising tool owing to its continuous monitoring capabilities, data analysis, and remote access features. ^33^ In 2024, a new device, QU-GM, was proposed, which is a continuous non-invasive device based on the end-to-end Internet of Things (IoT). The QU-GM device is composed of a PPG sensor, a microcontroller, and a battery. It is a BG measurement system that uses photoplethysmography (PPG) signals, blood pressure, and demographic information. After filtering the collected data, they are sent to the backend server and reported back to the mobile application. The device can be worn as a wrist watch. The authors checked the proposed model’s prediction and ensure that it is clinically acceptable, where K-nearest neighbor model classified the severity levels with an accuracy of 98.12%. Therefore, the wristbands that can be connected to an Android mobile application were deployed in Amazon Web Server. ^34^

Recently, IoT-enabled Continuous Glucose Monitors (IAI-CGM) framework has been used to understand user intentions to adopt Internet of Things (IoT)-enabled Continuous Glucose Monitors (CGMs). The framework was used to assess patient acceptance and use of these devices. It showed that patients need to have personal alarm systems, and IoT-CGMs have the potential to serve a useful role in the function of an alarm clock as well as provide real-time monitoring. The author concluded that in the domains of wearable technology and healthcare, the adoption of IoT-CGM is a crucial but still understudied issue. ^35^

IoT and the interconnected sensors and devices, provides real-time data from environment. From smartwatches that monitor heart rate and activity levels to continuous glucose monitors for diabetic patients, IoT devices can present an individual’s health status. A significant challenge is presented due to the huge volume and velocity of data generated by these devices present. This is where Artificial Intelligence (AI) algorithms are started to be part of the healthcare field. Therefore, machine learning and deep learning are with great ability to analyze these massive datasets, uncover hidden patterns, and generate actionable insights. ^36^

 In conclusion, many commercial devices are small, and lightweight, but may be visible to others. Another kind of devices either they are manually used or must be injected under the skin where its lifetime is limited, and needs to be replaced. Abbott Freestyle Library CGM with wireless sensor that replaced on the skin. It is suitable for all patient from age 4 years and can be interconnected with an available smartphone application since IoT for non-invasive BG is now involved too.

When we start this work, we observed that patients do not feel comfortable with the CGM alarm every 15 min, also, the limited data storage cannot help follow the patient’s health situation. Though, our goal is to overcome these two gaps using IoT technologies.

To support our study, we distributed a questionnaire with 11 questions to determine if patients were willing to change their attentions for testing their BG using the new technologies.

## III. Questionnaire

Monitoring of blood glucose levels in patients with diabetes is an important continuous procedure. BG monitoring using the traditional method (finger prick) causes patient discomfort, potential infection, and financial burden. For these reasons, the following thoughts became a must:

• Keeping the patient healthy almost all times without the need for extra assistance.
• Reduce pain and infections.
• Enable elder patients to make their health decisions at home and decrease the need to visit diabetic centers frequently.
  A questionnaire survey was distributed to 10 women with Iraqi type 2 diabetes on oral medicine, aged between 45 and 60 years. The goal is to obtain information about the daily behavior of diabetic patients in Iraq and to know if they have the desire to use new IoT technologies such as CGM devices with skin sensors. The shaded cells in Table 1 concentrate on the use of the new technology to test the BG level. The procedure first started by checking the glucose level using both the BGM by finger prick (traditional method) and the CGM sensor meter (using Abbott lifestyle device) at the same time. The questionnaire was then completed. Table 1 shows that patients with diabetes in Iraq depend on others in deciding their lifestyle and that they need to learn more about new technologies. The table shows that 9 out of 10 patients did not like finger prick testing, but 8 out of 10 patients felt uncomfortable using the CGM meter because they heard an alarm every 15 min. ^37^ This was the reason for starting this work because reducing the CGM device’s alarms while testing the BG level may change a patient’s mood and make him feel comfortable and willing to use CGM. The idea is to let the alarm work only when the BG is at a critical level (above 200 mg/dL or lower than 70 mg/dL), while the alarm stops when the BG is in the range (of 70–180 mg/dL). This enhancement can only be achieved by connecting CGM to a personal smart device.

**Table 1.** The questionnaire results after testing the glucose level using both the finger prick and a CGM sensor meter for 10 women with type 2 diabetes. ^37^

Question	Yes	No	Abstain
Do you trust your professional who help you to make your decision	9	1	-
Do you feel that diabetes affects every part of your life	10	-	-
Do you think that decision regarding daily diabetes care should be based only on finger prick	7	3	-
Do you ask the professional about the latest way to check the glucose level	2	8	-
Do you think that using two ways for testing glucose level is better than one	3	7	-
Do you feel that finger prick is painful and uncomfortable	9	1	-
Do you check the markets for new way to check glucose level	1	9	-
Would you trust sensor meter to be used daily for checking your glucose level	5	4	1
Do you feel uncomfortable with sensor meter that show you the result of glucose level every 15 min	8	2	-
Do you need more information about how sensor meter works	10	-	-
Would you change the way of checking your glucose level to be by sensor meter only	5	3	2
Total 100%	62.73%	34.54%	2.73%

## IV. Materials and methods

The work is based on the use of an available CGM device in Iraqi’s market with a reasonable price (e.g., the Abbott Freestyle Library) plus a smart device where most people can use such as personal mobile devices. The main idea is to enhance the comfort of the CGM device by connecting it to a program installed in a personal smart device. The suggested method is easy to understand, so a patient can depend on its output without the need for extra assistance. The CGM device can be connected to a personal smart device by using the Bluetooth. Moreover, this connection gives the ability to benefit from the cloud storage to store large BG test results, where the stored data can be used to calculate A1C and estimate A1C (eA1C) using GMI calculation. This means that the installed program algorithms are used to control the CGM alarms and to calculate A1C and GMI every two weeks based on the stored BG test results. If GMI is < 5.7%, means that the patient had a good lifestyle during these past two weeks.

### A. Materials

The Abbott Freestyle Library (CGM) was used to monitor the BG, where each sensor lifetime was approximately 14 days. This device can be used for patients aged ≥ 4 years because of its ease of use and can be interconnected with an available smartphone application. ^38^ The only restriction was the alarm sound every 15 min.

**Figure 6.**
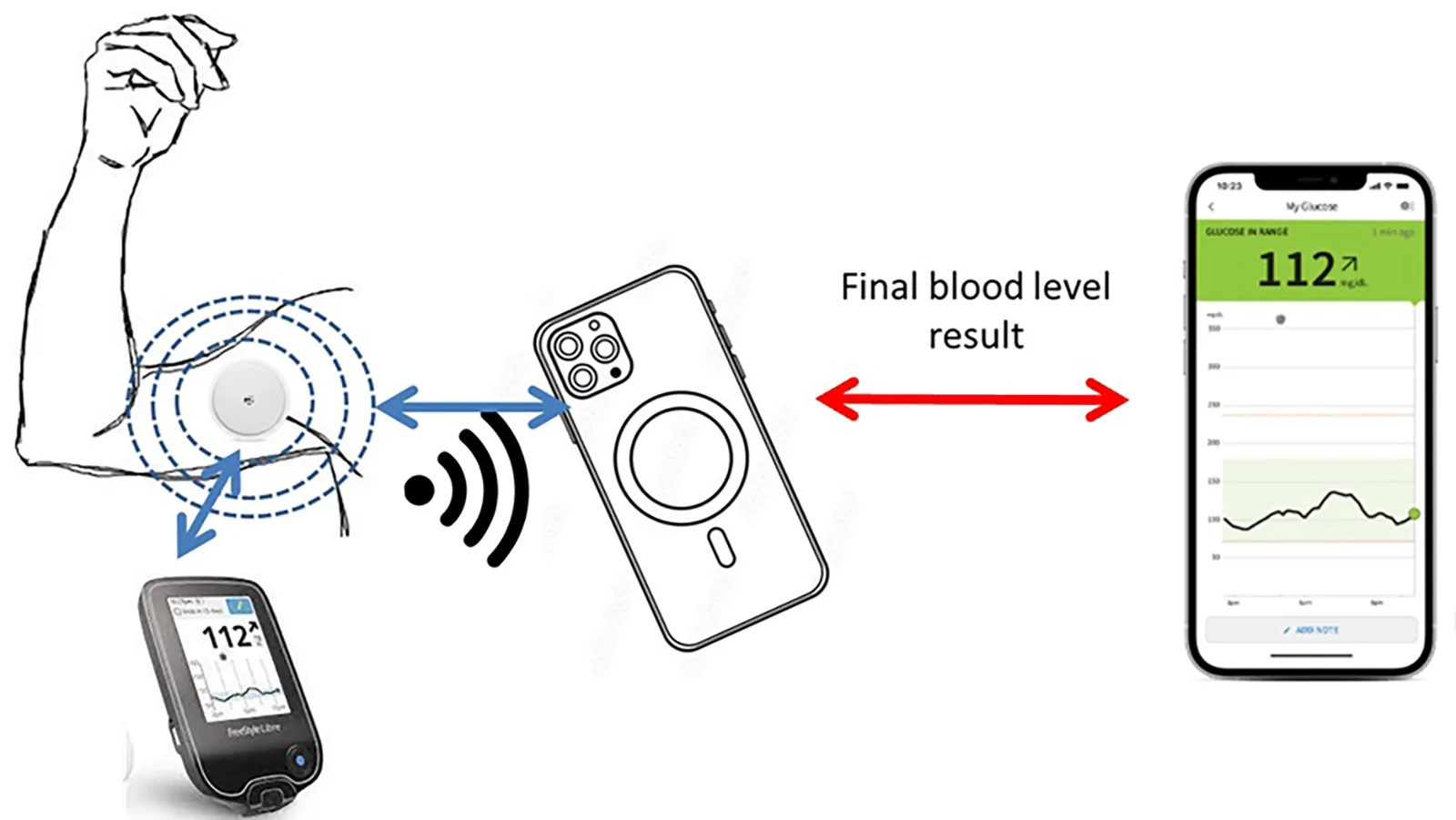
Abbott freestyle libre CGM and IoT technology.

The suggested model is considered a new way to let the patient know his health situation only when it is critical and needs further attention by connecting the CGM device to a smart device to avoid unnecessary alarms. For more benefit from the stored BG results in the CGM device, a model is created to calculate the A1C for the patient to let him know if he is in the correct track of his health lifestyle. The personal smart device uses the cloud for extra storage space to collect BG data in a special database (DB) for GMI calculation using data from the past two weeks. The sketch in Figure 6 shows the devices and the connections between them, while the entire algorithm steps are explained in the solution approach subsection.

### B. Ambulatory Glucose Profile (AGP)

“AGP is considered as the standardized, practical one page report for graphically presenting a summary of glycemic control status in patients with diabetes who use continuous glucose monitoring (CGM) systems as part of their daily diabetes care.” The AGP report consists of three sections.

**Figure 7.**
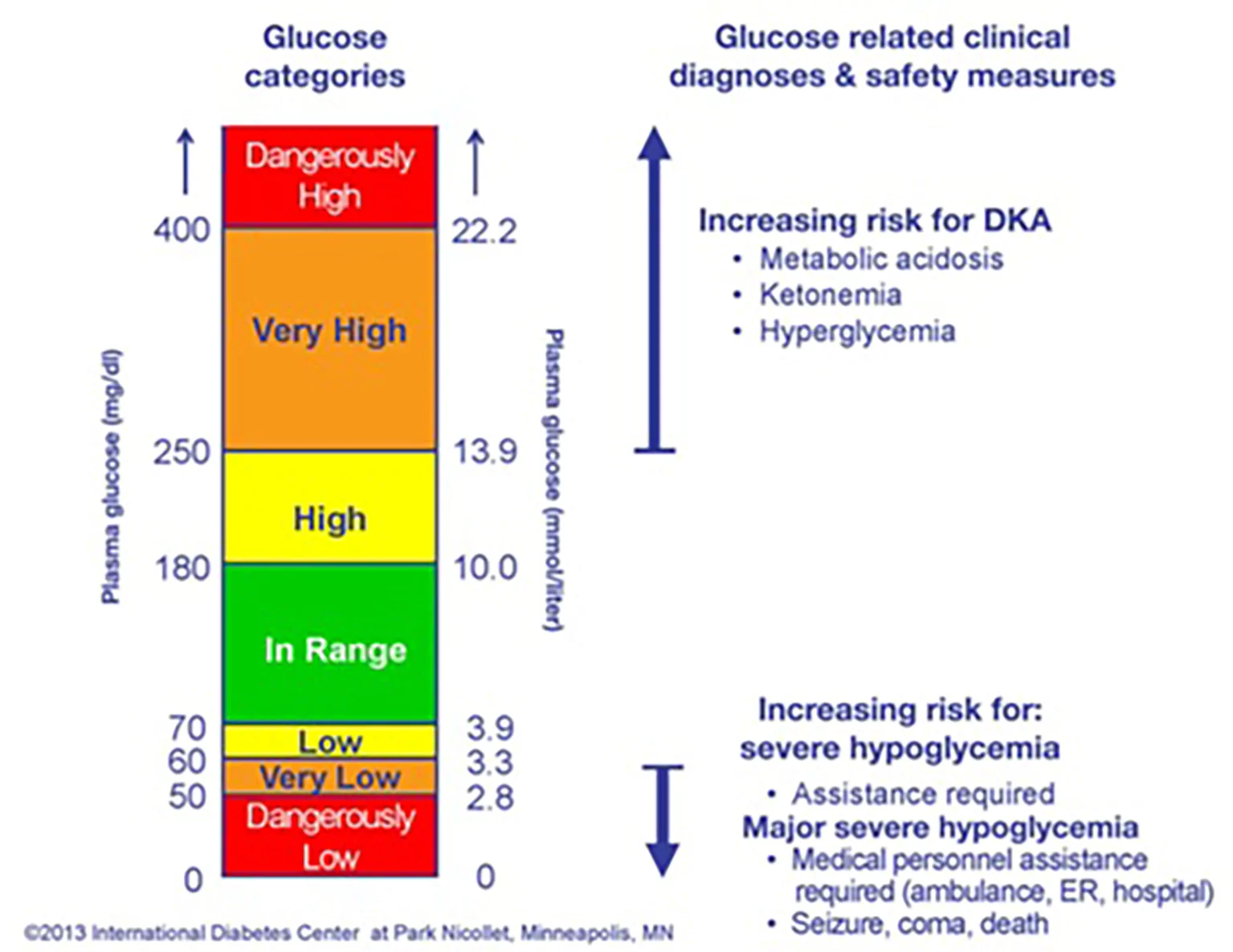
Glucose target ranges and categories. Dx, diagnosis; DKA, diabetic ketoacidosis; Glu, glucose; Hypo, hypoglycemia; Maj, major; ER, emergency room; admit, admittance. ^42^

(1) glucose statistics and targets, (2) ambulatory glucose profile, and (3) daily glucose profile. ^39, 40^ The International Diabetes Center (DIC) developed a data analysis software program for CGM that considers the time in range (TIR) over a period of time. TIR is a metric of glycemic control that provides more actionable information than A1C alone by establishing target percentages of time in different glycemic ranges to identify the needs of diabetic populations. The target is to have a TIR of 47% and a glucose range of 70–180 mg/d. ^39, 41^ A consensus-recommended AGP report for a given patient can be generated in LibreView on a cloud-based platform ( http://www.libreview.com). This platform is available for many countries but not for Iraq, which provides the idea for presenting this work to give Iraqi patients a way to manage their own health using this work algorithm that considers the international standardization AGP. AGP shows three levels of glycemic control: in the range between 70 mg/dl and 180 mg/dl, hyperglycemia is gradient from >180 mg/dl to >400 mg/dl (22.2 mmol/liter), while hypoglycemia is considered under 70 mg/dl (3.9 mmol/liter), as shown in Figure 7. ^42^

### C. Methods

Initially, we tested the efficiency of the Abbott freestyle library device by comparing its results with the standard finger-prick BG test for all 10 patients for 24 h. The finger-prick test was performed three times a day (before breakfast, after breakfast, and at night before sleeping), while CGM tests the BG every 15 min. The averages of both tests based on Equation 1 for the finger pick test and Equation 2 for the CGM test are presented in Tables 2 and 3, respectively. The results in Table 3 show the average of the test results every 8 h. ^37^

$$\mathrm{Avg}._{\mathrm{BG}}=\frac{{\text{BeforeBK}}_{\mathrm{BG}}+{\text{AfterBK}}_{\mathrm{BG}}+{\text{TwohBbed}}_{\mathrm{BG}}}{3}$$ (1)

$$\mathrm{Avg}._{\mathrm{BG}}=\frac{\sum_{i=1}^{n=96}x_{i}}{96}$$ (2)

**Table 2.** The finger prick test for 10 patients in 24 hours. ^37^

No.	Before BK (6 am-2 pm)	Immediately after BK	2 hours before bedtime	Total	SD
1control	90	95	90	91.6	20.223
2	128	159	121	136	18.876
3	92	117	129	112.6	22.722
4	144	189	161	164.6	23.430
5	121	160	118	133	16.441
6	205	180	174	186.3	13.428
7	114	135	110	119.6	24.826
8	132	175	132	146.3	47.077
9	150	238	165	184.3	35.679
10	200	270	247	239	10.263
11	146	166	152	154.6	20.223
Mean ± SD	143.2 ± 35.627	178.9 ± 45.447	150.9 ± 40.217	157.63 ± 37.84	

**Table 3.** The CGM test from Abbott freestyle libre for 10 patients in 24 hours. ^37^

No.	Avg. 6 am-2 pm	Avg. 2 pm-10 pm	Avg. 10 pm-6 am	Total	SD
1control	88	90.7	90	89.56	16.941
2	124.3	154.9	127	135.4	17.683
3	89.9	111.2	125	108.7	20.232
4	142	181.3	170	164.4	22.350
5	111.3	155	125	130.4	12.503
6	192	189	169	183.3	1.184
7	120	122.1	120.1	120.7	21.101
8	130	170.3	139.3	146.5	42.111
9	149	230.3	170.6	183.3	30.008
10	198	250.9	249	232.6	8.710
11	145.1	162.3	156.1	154.5	16.941
Mean ± SD	127.73 ± 35.319	157.24 ± 43.281	142.41 ± 38.801	155.98 ± 36.64	

where *x _i_* is the test value every 15 min for a day. The Statistical Package for the Social Sciences software (version 24.0) was used to perform all statistical analyses, normality of data, and parametric statistical tests.

The paired (dependent) t-test is used to assess the significance of differences between the two ways of measuring BG. The finding of the test is *t* = 2.274, with α = 0.05 and two-tailed test, the *t*_*critical*_ = 2.262 meaning that *t* > *t*_*critical*_. Therefore, the study demonstrated that, significantly at P < 0.05, there were no differences between both methods.

**Figure 8.**
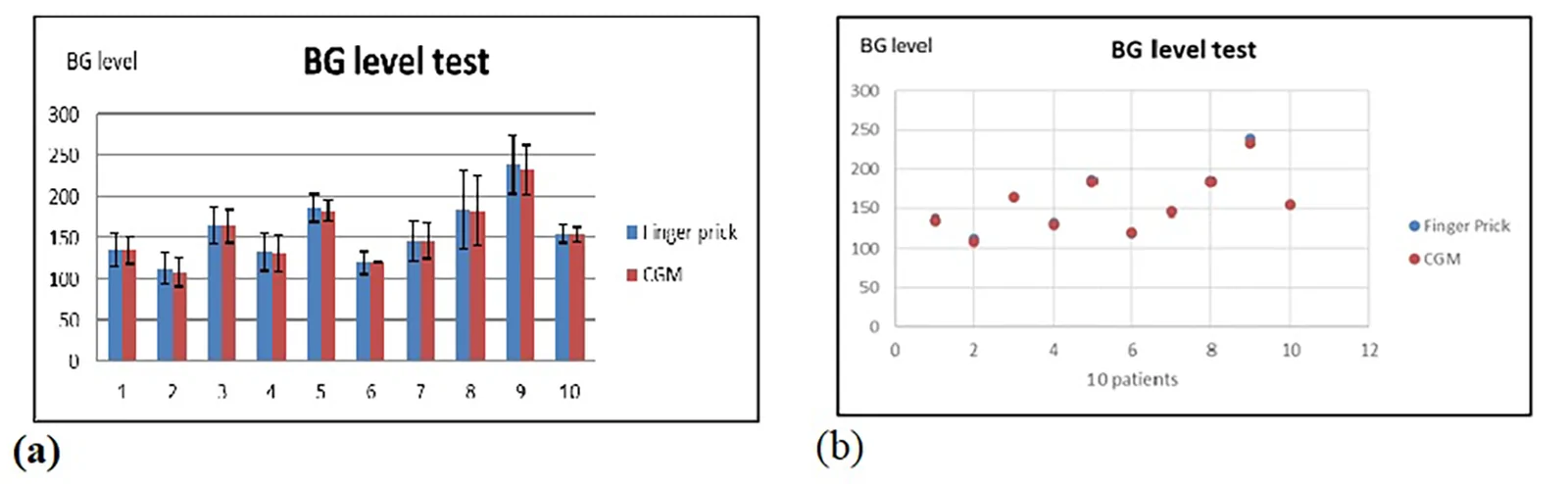
(a) Diagram (b) Scatter plot. Both shows the mean and standard deviation for finger prick and CGM tests for 10 patients in 24 hours. ^37^

Figure 8 shows the results of the comparison, where the data are almost similar for both tests, which means that the CGM device with the skin sensor that we used can measure blood glucose efficiently.

Usually, an A1C test is done every two or three months in the laboratory to check for improvement or worsening of BG; however, a significant change in BG can be known within two weeks. We used the stored data for 14 days to calculate A1C in our algorithm by using a standard formula to estimate A1C (eA1C) based on GMI, which gives an approximate value to the laboratory A1C.

The GMI is the average (mean) glucose value based on data collected by the CGM. The average glucose values from the CGM to obtain the GMI percentage can be calculated using Equation 3.

Both A1C (eA1C) and GMI provide information to maintain better control over blood sugar levels, and eA1C% can be obtained using Equation 4. The estimated A1C can then be calculated using Equation 5. ^43^

$$\mathrm{GMI}(\%)=3.32+0,02392[\text{meanCGMdata}(\frac{\mathrm{mg}}{\mathrm{dl}})]$$ (3)

$$\text{eAIC}(\%)=\frac{(\mathrm{GMI}(\frac{\mathrm{mg}}{\mathrm{dl}}))+46.7}{28.7}$$ (4)

$$\mathrm{AIC}(\frac{\mathrm{mg}}{\mathrm{dl}})=(10.929(\mathrm{AIC}(\%)-2.15))18.05$$ (5)

The term mean CGM data is the average glucose level over a specific period and can be found from Equation 6.

$$\text{Grand}\;\mathrm{Avg}._{\mathrm{BG}}=\sum_{j=1}^{14}{\left(\frac{\overset{n=96}{\underset{i=1}{\sum x_{i}}}}{96}\right)}_{j}$$ (6)

According to, ^44, 45^ the GMI and A1C may not agree if the patient has acute hyperglycemia due to illness, steroid administration, or diabetic ketoacidosis; thus, GMI will be higher than the laboratory A1C measured at the same time and vice versa, where the GMI is lower than A1C if the patient suffers from hypoglycemia. This fact is used to alert the patient with a colored alarm (red, yellow, or green) to indicate hyperglycemia, hypoglycemia, or stability.

Another idea suggested to be added to this work is the use of a Markov chain to predict the GMI and A1C for the next 14 days. This can be done based on the stored data of past GMI results to allow the patient to find a healthy lifestyle. Because CGM provides glucose readings every few minutes, from this data, a calculation is made to estimate the GMI for each day using Equation 3.

The Markov chain concept was adopted from a previous study. ^46^ Their idea was to collect sequential blood sugar measurements and define discrete states: hypoglycemic, normal, and hyperglycemic. A transition probability matrix (TPM) was used to show the likelihood of moving from one state to another. The model is used to predict or detect anomalies in blood sugar behavior.

The idea of our suggestion is to build a three-state ( *S _i_*, where *i* = 0 to 2) model, where state *S*_0_ indicates good control (stable in BG), while *S*_1_ and *S*_2_ indicate moderate and poor control, respectively. The intervals based on BG average are as: 7.3% < GMI < 7.8% for *S*_0_, 7.8% < GMI < 8.0% for *S*_1_, while GMI < 7.2% or GMI > 8.0% would be for *S*_2_.

The state of transition is a change from one state to another based on the next period, where this transition state is a random process and is expressed in the form of probability.

The suggested TPM is as follow:

$$P_{ij}=[\begin{array}{ccc} 0.8 & 0.1 & 0.1\\ 0.2 & 0.3 & 0.5\\ 0.3 & 0.1 & 0.6 \end{array}]$$ (7)

Where *P*_*ij*_ represents the probability of staying in the same state, while the probability of reaching state *j* from state *i* in one day is *P*_*ij*_.

To calculate the state distribution after 14 days, we used Equation 7, where $\pi_{0}$ represents the initial state vector. This gives the probability of being in each state after 14 days.

$$\pi_{14}=\pi_{0}\times P^{14}$$ (8)

In this way, an estimated result can be calculated to help the patient find if he is on the right track of his lifestyle or if the lifestyle needs some changes.

### D. Solution approach

This study aims to benefit from IoT technologies by connecting a CGM device to a personal smart device to transfer data between them and to be stored in a cloud database. Another benefit of this connection is that it enhances the CGM device based on the three proposed algorithms.

Algorithm 1 calculates the patient’s BG level based on AGP standardization, so that a message appears every 8 h to the smart device instead of the 15 min alarm during a stable health situation; otherwise, an emergency-colored message will appear with a ringing bell to alert the patient.

Algorithm 2 uses the data stored to calculate A1C every 14 days, based on the standard in Table 4. Then, a comparison between GMI and A1C will start, and the result is sent to the patient as a colored alarm either red, yellow, or green to indicate hyperglycemia, hypoglycemia, or being stable, respectively.

**Table 4.** A difference in laboratory-measured A1C and GMI and what it may mean. ^44^

A1C vs. GMI	
8.0% vs. 7.8%	A1C is like GMI means that the average CGM glucose level is about what would be predicted from the measured A1C. **(Green color)**
8.0% vs. 7.2%	A1C is higher than GMI means that the average CGM glucose level is lower than what would be predicted from the measured A1C. **(Yellow color)**
7.2% vs. 8.0%	A1C is lower than GMI means that the average CGM glucose level is higher than what would be predicted from the measured A1C. **(Red color)**

Algorithm 3 calculates the GMI every two weeks depending on the stored data, and a calculation is also performed to estimate the result for the next 14 days to help the patient to control his lifestyle. A suggested idea may be added to the algorithm to estimate A1C every 3 months for more accurate results based on the stored outputs.

The solution is based on Equation (2) to calculate the mean glucose of one day and on Equation (6) to obtain the mean CGM data, while Equations (4) and (5) are used to calculate the predicted GMI for the next 14 days based on the Markov chain model using three states, as mentioned before.

Algorithm 1. **Beginning the process in personal smart device.**

1: Receive BG level from CGM every *t* = 15 min, where (t) is the device alarm duration

2: Check BG level according to AGP

3: If 70 ≥ BG ≥ 180 then nothing happened go to step 4

  Else

   If 181 ≥ BG ≥ 400 then alarm the patient with red color

   Else Alarm the patient with yellow color

   Endif

  Endif

4: *t* = *t* + 15

5: If time *t* = 8 hours (480 min) go to step 6

  Else continue with step 1

  Endif

6: If t = 24 hours (1440 min) calculate *Avg. _BG_* for every day using Equation 2

7: Go to Algorithm 2

Algorithm 2. **Calculating A1C.**

1: When time t = 8 hours then alarm the patient about his situation

2: Counting days (j) for every *j = j* + 1

3: If *j <* 14 days then store data and go to Algorithm 1

  Else

   If *j* = 14 calculate A1C using Equations 5 and let j = 0

   Endif

  Endif

4: Calculate GMI% using Equation 3

5: Compare the A1C% result with GMI% according to Table 4

6: Show patient the result as a colored message *(either red, yellow or green)* on his smart device

7: Go to Algorithm 3

Algorithm 3. **Predicting GMI% for the next 14 days.**

1: Use the stored GMI% for the past 14 days

2: Processing Markov Chain with three stages to predict GMI

4: If the predicted GMI is red or yellow the patient needs to control his lifestyle

  Else (green) means that the patient is on the right track of lifestyle

  Endif

5: Counting days (k)

6: If k = 90 then recall data from the cloud database to calculate GMI% and Show result to patient with colored alarm

  let k = 0

  Else go to step 7

  Endif

7: Go to Algorithm 1

### E. Discussion

The proposed model intends to improve CGM to be more comfortable for diabetic patient. The suggested model integrates CGM data with three algorithms implemented on a patient’s smart device to support lifestyle management for individuals with type 2 diabetes. A historical CGM data stored in the cloud can be used to calculate the (GMI%) every two weeks and do an A1C test every two or three months. Markov chain can be used to predict GMI% for the following 14 days to provides an early indication of the patient’s lifestyle. The predictive capability is expected to help patients recognize patterns in their glucose levels and adjust their behavior accordingly. Next step will involve implementing the model on a smart device and validating its predictive accuracy using real patient data to quantitatively evaluate improvements in blood glucose management and user comfort.

## V. Conclusion

The work in this research discusses many subjects and can be divided into five goals: (1) First is to give an overview of non-invasive glucose level monitoring optical principles with the latest researches about it. Then, a summary of non-invasive glucose level monitoring technologies is provided. (2) To make this work a reference to the latest works in the field of diabetic diseases, where IoT technologies used to support patients individually. This done by reviewing many commercial CGM home devices with reasonable prices and granted by the WHO. Then a comparison between finger prick and CGM is done to show the CGM efficiency (all volunteers used Abbott Freestyle Libre CGM). (3) To study how Iraqi patients with type 2 diabetes feel when using a CGM device. A questionnaire was administered to 10 Iraqi women with type 2 diabetes and aged–45-60 years old. The questionnaire discussed and showed that diabetes patients in Iraq need more education about using new IoT technologies. (4) Suggesting a model with three algorithms that installed in the patient’s personal smart device and connected to a CGM device to control his/her own lifestyle. first algorithm enhances the CGM device by showing a message every 8 h in a normal situation rather than every 15 min to make it more comfortable. (5) CGM connected to smart device has the ability to store data in cloud storage, where the second algorithm uses this data to calculate GMI% every two weeks. Also, GMI% will be predicted for the next 14 days based on the Markov chain to indicates the lifestyle of the patient during the past period.

In the future, we plan to implement our model in a smart device to obtain real results so that we can prove the high benefit of this model in managing a patient’s BG efficiently and comfortably. In order to overcome the sudden blood sugar changes, such as nighttime hypoglycemia, a new idea is taken into consideration which is to connect a second smart device of a relative or a family doctor to the model. In this way this person can receive the same patient’s alert message at the same time.

Moreover, a suggested idea can be added to estimate A1C every 3 months using a machine learning algorithm for more accurate results based on the stored outputs.

## Ethical considerations

Ethical approval was obtained from the Ethics committee of University of Baghdad/Institute of Laser for Postgraduate Studies (Approval number: 576) signed by the chairman of the research ethics committee Prof. Dr. Abdulhadi Al-Janabi. All participants provided written informed consent after receiving a detailed explanation of the study purpose, procedures, potential risks, and benefits. Participants used CGM devices in their usual environments, such as their homes or offices, without the need to visit a laboratory or health center. All data were handled in accordance with the confidentiality and privacy guidelines.

## Data Availability

Repository name: Questionnaire survey and comparing between FP standard test and CGM test for BG. https://doi.org/10.5281/zenodo.17914313. ^37^ The project contains the following underlying data:
• questionnaire.csv (summery of the questionnaire with 11 questions for 10 patients answering raw with either yes, no, or abstain) • pair test.csv (Table-FP test raw, before breakfast, after breakfast, and before sleeping. Table-CGM test raw, Avg. 6am -2pm, Avg. 2pm – 10pm, and Avg. 10pm – 6am. Table-total Avg raw, and SD raw) All relevant data are included in the main manuscript and in the underlying dataset. **Questionnaire**. To determine whether patients are willing to change their attentions for testing their BG using new technologies). Data are available under the terms of the Creative Commons Zero “No rights reserved” data waiver (CC0 1.0 Public domain dedication).
