# Supplementary material for: Advancing Diabetic Care Through Non-Invasive Glucose Monitoring Using Optical Sensors and IoT Technologies
Source: F1000Res. 2026 May 5;15:108. Originally published 2026 Jan 23. [Version 2] doi: 10.12688/f1000research.173871.2 (PMC13554737; doi:10.12688/f1000research.173871.2)
Supplement: Peer review history

# Reviewer report: Reviewer response for version 2

**DOI:** 10.5256/f1000research.197253.r481843

**Published:** 2026-07-22

**Authors:** Israr Ahmed

**Affiliations:** 1 Khalifa University of Science and Technology, Abu Dhabi, United Arab Emirates

All of my comments have been addressed, and I have no new comments. Therefore, I would recommend indexing the article.

Is the work clearly and accurately presented and does it cite the current literature?

Partly

If applicable, is the statistical analysis and its interpretation appropriate?

Partly

Are all the source data underlying the results available to ensure full reproducibility?

Partly

Is the study design appropriate and is the work technically sound?

Yes

Are the conclusions drawn adequately supported by the results?

Yes

Are sufficient details of methods and analysis provided to allow replication by others?

Partly

Reviewer Expertise:

Optics; Optical Sensing; Glucose Sensing; Nanotechnology; 2D materials

I confirm that I have read this submission and believe that I have an appropriate level of expertise to confirm that it is of an acceptable scientific standard.

# Reviewer report: Reviewer response for version 2

**DOI:** 10.5256/f1000research.197253.r481842

**Published:** 2026-05-07

**Authors:** Mohammad Abdolrazzaghi

**Affiliations:** 1 University of Toronto, Toronto, Ontario, Canada

Thanks for applying the comments. No more comments are required.

Is the work clearly and accurately presented and does it cite the current literature?

Yes

If applicable, is the statistical analysis and its interpretation appropriate?

Yes

Are all the source data underlying the results available to ensure full reproducibility?

Partly

Is the study design appropriate and is the work technically sound?

Yes

Are the conclusions drawn adequately supported by the results?

Yes

Are sufficient details of methods and analysis provided to allow replication by others?

Yes

Reviewer Expertise:

glucose sensing, microwave sensors, machine learning

I confirm that I have read this submission and believe that I have an appropriate level of expertise to confirm that it is of an acceptable scientific standard.

# Response

**Published:** 2026-05-07

**Authors:** Hanaa Basheer

**Affiliations:** Photonics Unit, University of Baghdad Institute of Laser for Postgraduate Studies, Baghdad, Baghdad Governorate, Iraq

Dear Dr. Mohammad

 Thank you for your comments, they were very helpful for improving our manuscript 

 Regards

# Reviewer report: Reviewer response for version 1

**DOI:** 10.5256/f1000research.191724.r453450

**Published:** 2026-03-06

**Authors:** Israr Ahmed

**Affiliations:** 1 Khalifa University of Science and Technology, Abu Dhabi, United Arab Emirates

The manuscript presents a system integrating a Continuous Glucose Monitoring (CGM) device with a smart-device and cloud-based architecture, proposing three algorithms for alarm optimization, A1C/GMI estimation, and short-term prediction using a Markov chain model. The study also includes a small questionnaire-based evaluation and comparison between finger-prick and CGM measurements.

 The topic is relevant and timely, particularly in the context of IoT-enabled healthcare systems. However, several substantial concerns regarding novelty, methodological rigor, statistical validation, and predictive modeling need to be addressed before the manuscript can be considered for indexing.

 1. The resolution of Figures 2a and 2d is poor and the text within the figures is not clearly readable. Please provide higher-quality images.

 2. It would strengthen the manuscript to include a short concluding paragraph at the end of each major section (e.g., “Optical approaches for non-invasive glucose monitoring” and “Commercial devices”) summarizing the key outcomes and insights from the discussion.

 3. Some sections would benefit from deeper discussion and inclusion of additional recent references to provide a more comprehensive and balanced overview.

 4. When discussing commercial devices (e.g., QU-GM and others), additional technical details would be helpful, such as limitations, reported accuracy, clinical validation status, and cost considerations.

 5. In the statistical comparison between CGM and finger-prick methods, since measurements were obtained from the same patients, please clarify whether a paired statistical test would be more appropriate than an independent samples t-test.

 6. For the proposed Markov chain model, please provide the transition probability matrix and clarify how these probabilities were estimated.

 7. If available, please include quantitative validation or brief discussion of the predictive performance of the proposed model.

 8. The link provided in the data availability section is not valid.

Is the work clearly and accurately presented and does it cite the current literature?

Partly

If applicable, is the statistical analysis and its interpretation appropriate?

Partly

Are all the source data underlying the results available to ensure full reproducibility?

Partly

Is the study design appropriate and is the work technically sound?

Yes

Are the conclusions drawn adequately supported by the results?

Yes

Are sufficient details of methods and analysis provided to allow replication by others?

Partly

Reviewer Expertise:

Optics; Optical Sensing; Glucose Sensing; Nanotechnology; 2D materials

I confirm that I have read this submission and believe that I have an appropriate level of expertise to confirm that it is of an acceptable scientific standard, however I have significant reservations, as outlined above.

# Response

**Published:** 2026-03-09

**Authors:** Hanaa Basheer

**Affiliations:** Photonics Unit, University of Baghdad Institute of Laser for Postgraduate Studies, Baghdad, Baghdad Governorate, Iraq

Dear Dr. Israr

 Thank you for the valuable notes. We tried to give full answer for each one of them. Please check the highlighted paragraphs on the revised manuscript. Regards

 1. The resolution of Figures 2a and 2d is poor and the text within the figures is not clearly readable. Please provide higher-quality images.

**Answer:** the figures resolution is limited to 300dpi as the editor requested.

 2. It would strengthen the manuscript to include a short concluding paragraph at the end of each major section (e.g., “Optical approaches for non-invasive glucose monitoring” and “Commercial devices”) summarizing the key outcomes and insights from the discussion.

**Answer:** we added short conclusions as your advice at the end of each section.

 3. Some sections would benefit from deeper discussion and inclusion of additional recent references to provide a more comprehensive and balanced overview.

**Answer:** We started our work late-2024 and submit it mid-2025, so all references we include were up to date. Therefore, and as you asked in (comment 4), we added more information about the presented devices.

 4. When discussing commercial devices (e.g., QU-GM and others), additional technical details would be helpful, such as limitations, reported accuracy, clinical validation status, and cost considerations.

**Answer: **more details are added for QU-GM and for the IAI-CGM) theoretical framework. We added new reference published in 2025 to show how AI involves with the healthcare fields 

 5. In the statistical comparison between CGM and finger-prick methods, since measurements were obtained from the same patients, please clarify whether a paired statistical test would be more appropriate than an independent samples t-test.

**Answer: **Thank you to draw attention to this point. We used pair (dependent) t-test to check the comparison between CGM and finger-prick methods

 6. For the proposed Markov chain model, please provide the transition probability matrix and clarify how these probabilities were estimated.

**Answer:** We thought that TPM is not suitable to be mentioned without implementation but after your comment the suggested TPM is added now to the paper with probability clarifying.

 7. If available, please include quantitative validation or brief discussion of the predictive performance of the proposed model.

**Answer:** We added a new discussion subsection to perform our proposed model

 8. The link provided in the data availability section is not valid.

**Answer:** We create a new dataset with valid link. Please check the revised manuscript, reference number [36]

# Response

**Published:** 2026-04-02

**Authors:** Hanaa Basheer

**Affiliations:** Photonics Unit, University of Baghdad Institute of Laser for Postgraduate Studies, Baghdad, Baghdad Governorate, Iraq

Dear Dr. Israr

 Thank you for the valuable notes. We tried to give full answer for each one of them. Please check the highlighted paragraphs on the revised manuscript. Regards

 1. The resolution of Figures 2a and 2d is poor and the text within the figures is not clearly readable. Please provide higher-quality images.

**Answer:** the figures resolution is limited to 300dpi as the editor requested.

 2. It would strengthen the manuscript to include a short concluding paragraph at the end of each major section (e.g., “Optical approaches for non-invasive glucose monitoring” and “Commercial devices”) summarizing the key outcomes and insights from the discussion.

**Answer:** we added short conclusions as your advice at the end of each section.

 3. Some sections would benefit from deeper discussion and inclusion of additional recent references to provide a more comprehensive and balanced overview.

**Answer:** We started our work late-2024 and submit it mid-2025, so all references we include were up to date. Therefore, and as you asked in (comment 4), we added more information about the presented devices.

 4. When discussing commercial devices (e.g., QU-GM and others), additional technical details would be helpful, such as limitations, reported accuracy, clinical validation status, and cost considerations.

**Answer: **more details are added for QU-GM and for the IAI-CGM) theoretical framework. We added new reference published in 2025 to show how AI involves with the healthcare fields 

 5. In the statistical comparison between CGM and finger-prick methods, since measurements were obtained from the same patients, please clarify whether a paired statistical test would be more appropriate than an independent samples t-test.

**Answer: **Thank you to draw attention to this point. We used pair (dependent) t-test to check the comparison between CGM and finger-prick methods

 6. For the proposed Markov chain model, please provide the transition probability matrix and clarify how these probabilities were estimated.

**Answer:** We thought that TPM is not suitable to be mentioned without implementation but after your comment the suggested TPM is added now to the paper with probability clarifying.

 7. If available, please include quantitative validation or brief discussion of the predictive performance of the proposed model.

**Answer:** We added a new discussion subsection to perform our proposed model

 8. The link provided in the data availability section is not valid.

**Answer:** We create a new dataset with valid link. Please check the revised manuscript, reference number [36]

# Reviewer report: Reviewer response for version 1

**DOI:** 10.5256/f1000research.191724.r457645

**Published:** 2026-02-26

**Authors:** Mohammad Abdolrazzaghi

**Affiliations:** 1 University of Toronto, Toronto, Ontario, Canada

Dear authors,

 The idea presented in this manuscript shows promise and demonstrates potential. The writing is clear, with measurements aligning well with simulations and yielding good results. However, several aspects need further clarification:

1. Introduction needs to be improved with considering the following recent relevant work and compare their potential use as wearables for BG detection:
  - Reference 1
  - Reference 2
2. The three-state Markov chain model is central to Algorithm 3, yet the manuscript provides no actual transition probability matrix (TPM), no training data, and no validation against real patient trajectories. How were the state boundaries (e.g., 7.3%–7.8% for S₀) selected, what is the sensitivity of the 14-day prediction to these thresholds?
3. The proposed system suppresses CGM alarms for up to 8 hours when BG is in the 70–180 mg/dL range. Given that sudden blood sugar changes, such as nighttime hypoglycemia, can occur and resolve well within this window, what clinical safety analysis or risk assessment has been performed to justify this interval?
4. All three algorithms are presented only as pseudocode with no actual implementation on a smart device. Can the authors provide details on the intended software architecture (e.g., mobile OS, Bluetooth protocol version, cloud platform, data encryption), and discuss how practical issues such as Bluetooth disconnection, data synchronization latency, and battery consumption would be handled to ensure reliable real-world deployment?
5. The study cohort consists of only 10 women aged 45–60 with type 2 diabetes on oral medication, and no formal statistical power analysis is reported. How do the authors justify the generalizability of both the questionnaire findings and the CGM-vs-finger-prick comparison to a broader diabetic population (e.g., male patients, type 1 diabetes, insulin-dependent type 2, younger or older age groups), and what sample size would be required to achieve statistically meaningful conclusions at the reported significance level of P < 0.05?

Is the work clearly and accurately presented and does it cite the current literature?

Yes

If applicable, is the statistical analysis and its interpretation appropriate?

Yes

Are all the source data underlying the results available to ensure full reproducibility?

Partly

Is the study design appropriate and is the work technically sound?

Yes

Are the conclusions drawn adequately supported by the results?

Yes

Are sufficient details of methods and analysis provided to allow replication by others?

Yes

Reviewer Expertise:

glucose sensing, microwave sensors, machine learning

I confirm that I have read this submission and believe that I have an appropriate level of expertise to confirm that it is of an acceptable scientific standard, however I have significant reservations, as outlined above.

## References

[1] : Noninvasive Glucose Sensing in Aqueous Solutions Using an Active Split-Ring Resonator. *IEEE Sensors Journal* .2021;21(17) : 10.1109/JSEN.2021.3090050 18742-18755 10.1109/JSEN.2021.3090050

[2] : In–human testing of a non-invasive continuous low–energy microwave glucose sensor with advanced machine learning capabilities. *Biosensors and Bioelectronics* .2023;241: 10.1016/j.bios.2023.115668 10.1016/j.bios.2023.115668 37774465

# Response

**Published:** 2026-02-28

**Authors:** Hanaa Basheer

**Affiliations:** Photonics Unit, University of Baghdad Institute of Laser for Postgraduate Studies, Baghdad, Baghdad Governorate, Iraq

Dear **Mohammad Abdolrazzaghi**

 Thank you for the great advice that we had from your comments. Below our answers to them all

  

- Introduction needs to be improved with considering the following recent relevant work and compare their potential use as wearables for BG detection:

**Answer**: We did download the two papers and read them well. Both now are inserted in the introduction section with references number [15] and [16] when submitting the new draft

- The three-state Markov chain model is central to Algorithm 3, yet the manuscript provides no actual transition probability matrix (TPM), no training data, and no validation against real patient trajectories. How were the state boundaries (e.g., 7.3%–7.8% for S₀) selected, what is the sensitivity of the 14-day prediction to these thresholds?

**Answer**: The idea of using Markov chain is adopted from reference 43. We are intended to implement the model in our future work. The state boundaries are suggested according to table 4 and reference 40. We thought that TPM is not suitable to be mentioned without implementation but after your comment the suggested TPM is added now to the paper.

- The proposed system suppresses CGM alarms for up to 8 hours when BG is in the 70–180 mg/dL range. Given that sudden blood sugar changes, such as nighttime hypoglycemia, can occur and resolve well within this window, what clinical safety analysis or risk assessment has been performed to justify this interval?

**Answer:** this is a good point we just think about a colored alert message and a ringing bell but with your comment we will consider this important point by thinking about a second person such as a relative or a doctor to be aware of this situation immediately by sending him a copy of the alert message. This point is added as a future work to the paper. Thank you

- All three algorithms are presented only as pseudocode with no actual implementation on a smart device. Can the authors provide details on the intended software architecture (e.g., mobile OS, Bluetooth protocol version, cloud platform, data encryption), and discuss how practical issues such as Bluetooth disconnection, data synchronization latency, and battery consumption would be handled to ensure reliable real-world deployment?

**Answer:** Yes, we mention that as a future work

 (We plan to implement our model in a smart device to obtain real results so that we can prove the high benefit of this model in managing a patient’s BG efficiently and comfortably)

- The study cohort consists of only 10 women aged 45–60 with type 2 diabetes on oral medication, and no formal statistical power analysis is reported. How do the authors justify the generalizability of both the questionnaire findings and the CGM-vs-finger-prick comparison to a broader diabetic population (e.g., male patients, type 1 diabetes, insulin-dependent type 2, younger or older age groups), and what sample size would be required to achieve statistically meaningful conclusions at the reported significance level of P < 0.05?

**Answer:** I’m sorry I did not understand the comment well, but I will explain how our community think. The elderly patients in Iraq are stubborn especially with medicines and the way of treatment. They almost do not prefer any change or advise about new technology and this illustrated in table 1. The statistical Package for the Social Sciences software (version 24.0) was used to perform all statistical analyses, normality of data, and parametric statistical tests. The results are shown in table 2 and table 3.

# Response

**Published:** 2026-04-02

**Authors:** Hanaa Basheer

**Affiliations:** Photonics Unit, University of Baghdad Institute of Laser for Postgraduate Studies, Baghdad, Baghdad Governorate, Iraq

Dear **Mohammad Abdolrazzaghi**

 Thank you for the great advice that we had from your comments. Bellow our answers to them all

  

1. Introduction needs to be improved with considering the following recent relevant work and compare their potential use as wearables for BG detection:

**Answer**: We did download the two papers and read them well. Both now are inserted in the introduction section with references number [15] and [16]

1. The three-state Markov chain model is central to Algorithm 3, yet the manuscript provides no actual transition probability matrix (TPM), no training data, and no validation against real patient trajectories. How were the state boundaries (e.g., 7.3%–7.8% for S₀) selected, what is the sensitivity of the 14-day prediction to these thresholds?

**Answer**: The idea of using Markov chain is adopted from reference 43. We are intended to implement the model in our future work. The state boundaries are suggested according to table 4 and reference 40. We thought that TPM is not suitable to be mentioned without implementation but after your comment the suggested TPM is added now to the paper.

1. The proposed system suppresses CGM alarms for up to 8 hours when BG is in the 70–180 mg/dL range. Given that sudden blood sugar changes, such as nighttime hypoglycemia, can occur and resolve well within this window, what clinical safety analysis or risk assessment has been performed to justify this interval?

**Answer:** this is a good point we just think about a colored alert message and a ringing bell but with your comment we will consider this important point by thinking about a second person such as a relative or a doctor to be aware of this situation directly by sending him a copy of the alert message. This point is added as a future work to the paper. Thank you

1. All three algorithms are presented only as pseudocode with no actual implementation on a smart device. Can the authors provide details on the intended software architecture (e.g., mobile OS, Bluetooth protocol version, cloud platform, data encryption), and discuss how practical issues such as Bluetooth disconnection, data synchronization latency, and battery consumption would be handled to ensure reliable real-world deployment?

**Answer:** Yes, we mention that as a future work

 (We plan to implement our model in a smart device to obtain real results so that we can prove the high benefit of this model in managing a patient’s BG efficiently and comfortably)

1. The study cohort consists of only 10 women aged 45–60 with type 2 diabetes on oral medication, and no formal statistical power analysis is reported. How do the authors justify the generalizability of both the questionnaire findings and the CGM-vs-finger-prick comparison to a broader diabetic population (e.g., male patients, type 1 diabetes, insulin-dependent type 2, younger or older age groups), and what sample size would be required to achieve statistically meaningful conclusions at the reported significance level of P < 0.05?

**Answer:** I’m sorry I did not understand the comment well, but I will explain how our community think. The elderly patients in Iraq are stubborn especially with medicines and the way of treatment. They almost do not prefer any change or advise about new technology and this illustrated in table 1. The statistical Package for the Social Sciences software (version 24.0) was used to perform all statistical analyses, normality of data, and parametric statistical tests. The results are shown in table 2 and table 3.
